# Use of trachea-bronchial swab qPCR testing to confirm *Mycoplasma hyopneumoniae* seropositivity in an SPF breeding herd

**DOI:** 10.1186/s40813-018-0088-3

**Published:** 2018-06-01

**Authors:** Frédéric Vangroenweghe, Eveline Willems, Jiří Malášek, Olivier Thas, Dominiek Maes

**Affiliations:** 1 Elanco Animal Health Benelux, Benelux, Plantijn en Moretuslei 1 – 3rd floor, 2018 Antwerpen, Belgium; 2 Topigs-Norsvin International, Vught, The Netherlands; 3 Topigs Norsvin CZ, Brno, Czech Republic; 4 Department of Data Analysis and Mathematical Modelling, Faculty of Bioscience Engineering, Ghent University, Ghent, Belgium; 5 National Institute for Applied Statistics Research Australia (NIASRA), University of Wollongong, Wollongong, NSW Australia; 6 Department of Swine Herd Health and Reproduction, Faculty of Veterinary Medicine, Ghent University, Merelbeke, Belgium

## Abstract

**Background:**

A dedicated program to monitor for freedom of several economically important diseases is present within most of the breeding companies that currently deliver high health breeding animals to their customers. Serology is therefore the preferential approach in order to screen for most of these diseases, including *Mycoplasma hyopneumoniae* (*M. hyopneumoniae*). However, in case of positive serology, further decisions on farm health status and the related consequences should be based on additional confirmation tests.

**Case presentation:**

The current case report demonstrates that tracheo-bronchial swab (TBS) sampling is a suitable alternative to confirm a suspect *M. hyopneumoniae*-seropositive situation. A Central-European SPF herd was shown positive (90% positive, 10% suspect; *n* = 10) for *M. hyopneumoniae* using the conventional ELISA serology (Idexx HerdChek Mhyo ELISA) and a second ELISA test (IDEIA™ *Mycoplasma hyopneumoniae* EIA kit) did not exclude potential *M. hyopneumoniae* infection (10% positive, 70% suspect; n = 10). Further follow-up remained inconclusive on both tests. Throughout the entire monitoring period of 6 months, no coughing, necropsy lesions or lesions at slaughter could be detected which could confirm the *M. hyopneumoniae* health status. TBS sampling was used to confirm the health status for *M. hyopneumoniae*. In total, 162 samples were collected at different ages (*n* = 18 per age category): piglets at 3–6–9-12 and 15 wks of age, rearing gilts at 18–21-24 and 27 weeks of age. Collected TBS samples were negative for *M. hyopneumoniae* until 15 wks of age, but rearing gilts were highly *M. hyopneumoniae*-positive from 18 wks onwards with 87–100% *M. hyopneumoniae*-positive animals and PCR Ct-values between 25 and 33.

**Conclusions:**

This case report shows that collection of TBS samples to confirm the *M. hyopneumoniae* infection status of a breeding herd was able to provide additional information to serology in order to make crucial decisions concerning health management and eradication strategies within the breeding herd.

## Background

*Mycoplasma hyopneumoniae* (*M. hyopneumoniae)*, the primary pathogen of enzootic pneumonia, occurs worldwide and causes major economic losses to the pig industry. The pathogen adheres to and damages the ciliated epithelium of the respiratory tract. Affected pigs usually show chronic coughing, are more susceptible to other respiratory infections and have a reduced performance [1]). Moreover, *M. hyopneumoniae* plays a key role in the Porcine Respiratory Disease Complex (PRDC) through interactions with several other respiratory pathogens.

Piglets can become infected with *M. hyopneumoniae* during the suckling period and many studies have shown *M. hyopneumoniae*-positive animals from weaning onwards [2–7]. Moreover, once infected with *M. hyopneumoniae*, animals can excrete the pathogen over a long period of time, with total clearance lasting till 254 days post-infection [8]. This implies that infected gilts could carry *M. hyopneumoniae* well across their first pregnancy into their first lactation cycle, infecting their offspring with *M. hyopneumoniae* in early life.

Therefore, dedicated programs to monitor for freedom of *M. hyopneumoniae* have been developed within breeding companies that currently deliver high health breeding animals to their customers. Serology using ELISA is the preferential approach in order to screen for *M. hyopneumoniae* [9–14]. In case of positive serology, further decisions on farm health status and the related consequences should be based on additional confirmation tests. Clinical diagnosis of enzootic pneumonia can be verified by serological analysis [10]. However, in SPF programmes, the herd prevalence of *M. hyopneumoniae* infections is often low and the positive herd predictive value of a serological result decreases progressively with the decreasing herd prevalence [15]. Moreover, ELISA testing of sera from naturally infected pigs does not detect early-stage infection prior to seroconversion [16, 17], and infection and vaccination responses are indistinguishable. Under field conditions, the mean time to onset of coughing following an *M. hyopneumoniae* infection was 13 days, whereas the mean time between onset of coughing and seroconversion as measured by ELISA was 9 days [10]. Recent research has shown that currently used ELISA tests only start showing a seroconversion from 21 days post-infection onwards [18]. The percentage of animals seroconverting in the early stages of *M. hyopneumoniae* infection using one of the commercially available *M. hyopneumoniae* ELISAs remains relatively low (16–22% at 21 days and 35–45% at 28 days post-infection) [18]. This implies that a large number of samples is needed to reliably detect the presence of *M. hyopneumoniae* within the monitored herd. In the Danish SPF program, the final verification of herd infection with *M. hyopneumoniae* is consequently performed by demonstration of the agent [10]. A recent comparative study on diagnostic sampling approach for *M. hyopneumoniae* detection showed that laryngeal swabs were a reliable option to establish early detection of *M. hyopneumoniae*, followed by brocho-alveolar lavage fluids and nasal swabs [18]. Other innovative sampling techniques, such as tracheo-bronchial swab (TBS) sampling [6, 7, 19] have been introduced in combination with PCR detection of *M. hyopneumoniae* to reliably detect the pathogen of infected animals. The objective of the current case report is to show that TBS sampling is a suitable method to confirm a suspect *M. hyopneumoniae*-seropositive situation.

## Case presentation

### Case description

A high health breeding farm in Central Eastern Europe (220-sow herd) had been negative for *M. hyopneumoniae* for more than 20 years using a standard serological monitoring schedule (3×/year; 25 samples per time point) with a commercially available *M. hyopneumoniae* ELISA test (Idexx HerdChek Mhyo ELISA, indirect ELISA; Idexx Laboratories). Besides freedom for *M. hyopneumoniae*, the farm was also negative for *Pasteurella multocida* DNT+, *Sarcoptes scabiei* var. *suis*, *Brachyspira hyodysenteriae*, Porcine Reproductive and Respiratory Syndrome Virus (PRRSV) and *Actinobacillus pleuropneumoniae*.

External biosecurity is at the highest level, with no entrance to visitors and strict shower protocols for all farm personnel upon entrance of the farm. Internal biosecurity is also well established with boot hygiene (washing and disinfection) between production groups (sows, piglets, rearing gilts), clean disinfection baths at entrance of each individual compartment and no movement of other materials (cleaning equipment, pig handling materials, etc.) between production groups.

The sow farm is run on a 3-week batch management system with 7 groups of 32 sows each. Productive sows, weaned piglets and rearing gilts are housed in separate buildings on the premises. Successive batches of weaned piglets from 4 weeks until 16 weeks of age are housed in separate nursery compartments with strict all-in all-out (AI/AO) strategies. From 16 weeks of age onwards, rearing gilts are housed in a larger barn that is not managed according to AI/AO strategies.

### Standard serological monitoring for *M. hyopneumoniae*

At gilt delivery (27 weeks of age), regular serological sampling to assess *M. hyopneumoniae* status was performed throughout the last decade, repeatedly confirming the *M. hyopneumoniae*-negative status. The farm first tested positive for *M. hyopneumoniae* using the first ELISA serology (Idexx HerdChek Mhyo ELISA, indirect ELISA; Idexx Laboratories) in March 2017. Additional monitoring one month later (Idexx) confirmed the *M. hyopneumoniae* positivity and therefore, a second ELISA test (IDEIA™ *Mycoplasma hyopneumoniae* EIA kit; Oxoid – Thermo Scientific) was performed, demonstrating a clear evolution towards lower *M. hyopneumoniae*-positivity in both ELISA tests used. This decreasing trend did however not persist in the fourth sampling showing again a gradual increase in *M. hyopneumoniae*-titers. The number of positive samples obtained with both ELISA tests on each sampling date are given in Table 1.

**Table 1:** *Mycoplasma hyopneumoniae* standard ELISA (Idexx HerdChek Mhyo ELISA, indirect ELISA; Idexx Laboratories; S/P-ratio, sample to positive ratio) monitoring results from March 2017 onwards. Results of the second ELISA test (IDEIA™ *Mycoplasma hyopneumoniae*; Oxoid – Thermo Scientific; PI, percentage of inhibition) are also given. Table demonstrates total number of samples and number of samples with negative (Idexx, S/*P* < 0.30; IDEIA™, PI ≥ 65%), suspect (Idexx, 0.30 ≤ S/*P* ≤ 0.40; IDEIA™, 50% ≥ PI > 65%) or positive (Idexx, S/*P* > 0.40; IDEIA™, PI < 50%) *M. hyopneumoniae* ELISA results. Mean titers (± SEM) for Idexx ELISA (expressed as S/P-ratio) and IDEIA™ ELISA (expressed as PI) are given per sampling timepoint

Date	Analytical test	Analytical result			Results (mean ± SEM)	Total sample number
		Negative	Suspect	Positive		
20.3.2017	Idexx	0	1	9	0.86 ± 0.15	10
	IDEIA™	2	7	1	59.7 ± 7.7%	10
21.4.2017	Idexx	11	7	7	0.43 ± 0.08	25
	IDEIA™	17	4	4	65.4 ± 14.3%	25
25.4.2017	Idexx	18	0	4	0.17 ± 0.04	22
	IDEIA™	4	0	0	90.3 ± 15.0%	4
9.8.2017	Idexx	11	1	8	0.36 ± 0.06	20
	IDEIA™	2	0	2	58.0 ± 11.8%	4

### Monitoring of clinical signs and lung lesions

Moreover, throughout the entire monitoring period, no coughing, lung lesions at necropsy or at slaughter could be detected.

### Epidemiological information: *M. hyopneumoniae* in neighboring farms and wind direction

Several other swine farms, some belonging to the same production group, are located within a range of 2–3 km from the described SPF farm. Farm VS, a fattening unit directly related to the SPF source farm, is located at 1.35 km air distance in western direction and Farm VH, an unrelated 35-sow herd, is located at 2.25 km air distance in south-south-eastern direction. Epidemiological information on disease state, including *M. hyopneumoniae*, is actively exchanged among these different farms. Under the local conditions, wind direction is most often from a western direction with high wind speed (source: http://oze.tzb-info.cz/vetrna-energie/9800-vetrne-podminky-v-ceske-republice-ve-vysce-10-m-nad-povrchem-ii). During the last quarter of 2017, both farms (VS, fattening unit and VH, sow unit) were detected *M. hyopneumoniae*-positive on serological monitoring using the conventional ELISA test (Idexx HerdChek Mhyo ELISA, indirect ELISA; Idexx Laboratories).

### Diagnostic approach with TBS

Tracheo-bronchial sampling was performed as previously described [6, 7]. Briefly, TBS samples were obtained following restraint of the piglets with a nose snare, and subsequent use of a mouth opener. The aspiration tube used (CH12 × 50 cm; Medinorm) was inserted through the mouth and glottis down to the trachea-bronchial bifurcation where mucus was collected through gentle swab movement. The tip of the swab was collected in a sterile 10 mL polystyrene tube (MLS), mixed with 1 mL sterile saline and kept at 3–5 °C until analysis within 48 h of sampling.

### Analysis of tracheo-bronchial swabs

The material collected by TBS was processed in a *M. hyopneumoniae* p183 real-time-PCR [20]. Nucleic acid was extracted from TBS using an RNA/DNA isolation kit (MagMAX Pathogen RNA/DNA Kit; Life Technologies) and an automated nucleic acid isolation processor (MagMAX Express 96 processor; Life Technologies) based on magnetic bead technology. One millilitre of TBS was centrifuged for 5 min at 16,000 *g*, the pellet suspended in 400 μL lysis buffer, and 400 μL of the suspension was used as the sample. If no pellet was observable, 300 μL of the TBS was used as the sample. Bead mix and lysis/binding solution were added and the mix transferred onto a 96-well plate in the processor. Nucleic acid isolation was performed according to the manufacturer’s instructions. The PCR results were reported as negative (Ct ≥ 37) or positive (Ct < 37) for the presence of *M. hyopneumoniae* based on a Ct-threshold value. The detection limit range for *M. hyopneumoniae* reported [20] was from 10 ng/μL to 2.5 fg/μL. The detection limit for the PCR was validated for TBS spiked with dilutions of *M. hyopneumoniae* strain J (ATCC 25934) of at least 5 fg/μL.

### Sampling for determination of freedom of disease

The minimal number of samples needed to show ‘freedom of disease’ in the farm was calculated (http://epitools.ausvet.com.au), using FreeCalc – sample size calculation for freedom testing with imperfect tests using the modified hypergeometric distribution for exact hypothesis testing, with the assumption that maximum 2.5% of the animals (*n* = 6000) present on the farm were positive for *M. hyopneumoniae,* assuming test sensitivity and specificity of the TBS/qPCR combination of 75 and 100%, respectively [19, 20]. Based on this calculation, a total of 162 samples were collected at different ages (*n* = 18 per age category): piglets at 3–6–9-12 and 15 weeks of age and rearing gilts at 18–21-24 and 27 weeks of age.

### Statistical analysis for TBS positivity and Ct value

For the assessment of the overall effect of sampling date on the probability of a positive qPCR result, a Pearson chi-squared test was used. Pairwise comparisons between sampling dates was also performed with Pearson chi-squared tests, with *p*-values adjusted with the Holm procedure. All per-comparison p-values were computed by referring to the permutation null distribution of the test statistics. The latter were approximated based on 2000 random permutations. The overall effect of age (*n* = 9 age groups) on the average Ct values was assessed by means of an F-test in a one-way ANOVA. Post-hoc multiple comparisons of means were performed with Tukey’s method. The overall effect of age (*n* = 9 age groups) on the probability of a positive qPCR result was assessed by means of the Pearson chi-squared test. Multiple pairwise comparisons were also done with Pearson chi-squared tests, with p-values adjusted with Holm’s procedure.

All overall tests were performed at the 5% level of significance. Multiple comparison tests were performed simultaneously at the 5% familywise error rate (FWER) level. All data analyses were performed with the R statistical software version 3.4.3. [21].

## Results

### *M. hyopneumoniae* detection using TBS and subsequent PCR

**Fig. 1:**
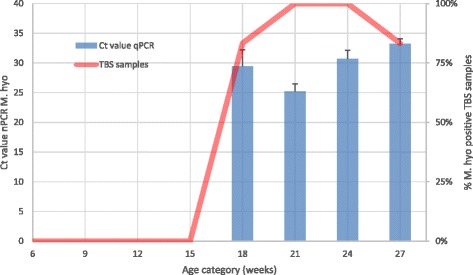
Tracheo-bronchial swab (TBS) sampling results of different age categories (*n* = 18 animals per age category) at the Central-European SPF herd. Piglets of 3–6–9-12 and 15 weeks and rearing gilt of 18–21-24 and 27 weeks of age were sampled. The qPCR *M. hyopneumoniae-*positive animals per age category were expressed as percentage (red line). The Ct values of those qPCR *M. hyopneumoniae*-positive TBS samples (Ct < 37) were expressed as means (± SEM; blue bars)

During lactation and nursery phase (from 3 until 15 weeks of age), no *M. hyopneumoniae* could be detected using PCR testing. However, once the animals entered the rearing barn located at the same site, *M. hyopneumoniae* was clearly present. Among the gilts from 18 until 27 weeks of age, the percentage of positive animals significantly increased (*P* < 0.05) to 87–100% in the oldest age group (Fig. 1). PCR Ct values initially decreased with age, starting at 29 in animals of 18 weeks of age, to 25 in animals of 21 weeks of age; and subsequently increased up to 30 and 33 in animals of 24 and 27 weeks of age, respectively (Fig. 1). Ct values were significantly different (*P* < 0.05) from 37, the cut-off value for PCR test positivity.

## Discussion

The current case report demonstrated that diagnosis of an *M. hyopneumoniae* infection can be difficult using the standard monitoring tools, such as clinical observation, serology [10] and slaughterhouse checks for typical lung lesions [1]. It was also shown that PCR testing on TBS samples confirmed the *M. hyopneumoniae* health status of a breeding herd. This allowed the farmer to make decisions concerning health management within the breeding company.

Interestingly, the collected TBS samples only showed *M. hyopneumoniae*-positive results from 18 weeks onwards, which is the age category that enters the gilt rearing barn. Until now, it is unclear how the *M. hyopneumoniae* infection has entered the farm. Generally, the major source of *M. hyopneumoniae* introduction into a farm is considered newly arrived subclinically *M. hyopneumoniae*-infected gilts, especially since *M. hyopneumoniae*-infected animals can excrete the pathogen for up to 254 days following initial infection [8]. However, this nucleus herd has not received any live breeding animals for many years.

A second hypothesis is that the *M. hyopneumoniae* entered the farm by airborne transmission from infected neighboring farms. Under favorable climatic conditions, aerogenous spread of *M. hyopneumoniae* between neighboring herds over varying distances has been demonstrated [22–24]. Indeed, the farm in the reported case had neighboring swine herds within a 1.5 to 3-km range. Moreover, epidemiological information shared among these neighboring farms clearly indicated that a break in *M. hyopneumoniae* health status had also happened during this year based on the results of their regular serological monitoring. Unfortunately, we do not have details on which of these swine farms first broke with its *M. hyopneumoniae* health status, although based on prevailing wind direction, farm VS which is located west from the SPF nucleus herd could be a good candidate.

Another source of potential introduction might be through an *M. hyopneumoniae*-infected transport vehicle. Especially under winter conditions, cleaning and disinfection of transport vehicles is not always easy to perform. Recently, *M. hyopneumoniae* survival on stainless steel at 4 °C was demonstrated for at least 2 days (max. 8 days for some strains) [25]. This would imply that the regular procedure for SPF pig-free downtime of 48 h for transport vehicles cannot always guarantee a 100% *M. hyopneumoniae*-free vehicle. Analysis of the *M. hyopneumoniae* infection kinetics on the farm revealed that the *M. hyopneumoniae* infection was first detected within the gilt rearing barn. This is also the location from which gilts are selected and loaded for external transport. Therefore, the probability of a biosecurity breach at the gilt loading point seems possible, since external biosecurity at all other levels has always been at the very high level. Moreover, once the *M. hyopneumoniae* infection entered this section of the farm, *M. hyopneumoniae* could easily further spread within the age groups from 18 until 27 weeks, which is not managed under strict AI/AO conditions. The reason why no coughing nor typical lung lesions were present is not clear. It may be due to the rather good housing conditions, e.g. air quality and level of dust [26, 27] and/or the fact that a low-virulent strain was circulating [28].

Although a break in biosecurity seems unlikely, it should be considered as another option for *M. hyopneumoniae* introduction into the farm. A 4-year research demonstrated that a 1-night downtime period is sufficient to prevent mechanical spread of both PRRSV and *M. hyopneumoniae* by personnel and fomites [29]. Moreover, that study concluded that basic sanitation procedures, such as hand hygiene and the use of both boots and coveralls should be enough to prevent mechanical spread and therefore, the implementation of a shower protocol is even not necessary [29]. In the present breeding farm, besides regular personnel, showering in on every entry, no visitors were admitted, except for the TBS sampling needed to confirm the *M. hyopneumoniae* infection status.

Further diagnostics is ongoing to confirm the *M. hyopneumoniae* infection status of the sow population, since TBS could not detect *M. hyopneumoniae*-positive piglets till 15 weeks of age. Based on the results of this monitoring, a specified eradication plan will be designed to result in a renewed *M. hyopneumoniae*-negative gilt outflow to the end customers.

## Conclusions

The present case report showed that for several months, *M. hyopneumoniae* infection in an SPF-herd may occur without clinical symptoms and typical lung lesions. In addition, serological testing may be difficult to interpret, and PCR testing on TBS may be needed to establish a conclusive diagnosis.

## Abbreviations

AI/AO: All-in/all-out
ANOVA: Analysis of variance
Ct value: threshold value
DNT: Dermo-necrotic toxin
ELISA: Enzyme-linked immunosorbent assay
*M.hyopneumoniae*: *Mycoplasma hyopneumoniae*
PCR: Polymerase chain reaction
PRRSV: Porcine Reproductive and Respiratory Syndrome Virus
qPCR: quantitative PCR
SPF: Specific pathogen free
TBS: Tracheo-bronchial swab
